# High density of CXCL12-positive immune cell infiltration predicts chemosensitivity and recurrence-free survival in ovarian carcinoma

**DOI:** 10.1007/s00432-023-05466-8

**Published:** 2023-11-15

**Authors:** Philipp Köhn, Alexandros Lalos, Alberto Posabella, Alexander Wilhelm, Athanasios Tampakis, Ercan Caner, Uwe Güth, Sylvia Stadlmann, Giulio C. Spagnoli, Salvatore Piscuoglio, Sabine Richarz, Tarik Delko, Raoul A. Droeser, Gad Singer

**Affiliations:** 1 University Center for Gastrointestinal and Liver Diseases (Clarunis), University of Basel, Spitalstrasse 21, 4031 Basel, Switzerland; 2 Institute of Pathology, University Hospital Basel, Schönbeinstrasse 40, 4031 Basel, Switzerland; 3 Brustzentrum Zürich, Seefeldstrasse 214, 8008 Zurich, Switzerland; 4 Department of Gynecology and Obstetrics, University Hospital Basel, Spitalstrasse 21, 4031 Basel, Switzerland; 5 Institute of Pathology, Kantonsspital Baden AG, Im Ergel 1, 5404 Baden, Switzerland; 6 Istituto CNR “Translational Pharmacology”, Rome, Italy; 7 Department of Biomedicine, University Hospital Basel, 4031 Basel, Switzerland; 8 Department of Vascular Surgery and Transplantation, University Hospital Basel, Spitalstrasse 21, 4031 Basel, Switzerland; 9 Chirurgie Zentrum Zentralschweiz/Surgical Center Central-Switzerland, Ärztehaus, St. Anna-Strasse 32, Lützelmatt 1, 6006 Luzern, Switzerland; 10 University of Basel, Petersgraben 4, 4031 Basel, Switzerland

**Keywords:** Ovarian carcinoma, CXCL12, Tissue microarray, Immunohistochemistry, Prognosis, Biomarker, Recurrence

## Abstract

**Background:**

Ovarian carcinoma is the most lethal gynecologic malignancy because of its late diagnosis, extremely high recurrence rate, and limited curative treatment options. In clinical practice, high-grade serous carcinoma (HGSC) predominates due to its frequency, high aggressiveness, and rapid development of drug resistance. Recent evidence suggests that CXCL12 is an important immunological factor in ovarian cancer progression. Therefore, we investigated the predictive and prognostic significance of the expression of this chemokine in tumor and immune cells in patients with HGSC.

**Methods:**

We studied a cohort of 47 primary high-grade serous ovarian carcinomas and their associated recurrences. A tissue microarray was constructed to evaluate the CXCL12 immunostained tumor tissue. CXCL12 expression was evaluated and statistically analyzed to correlate clinicopathologic data, overall survival, and recurrence-free survival.

**Results:**

A high proportion of CXCL12 + positive immune cells in primary ovarian serous carcinoma correlated significantly with chemosensitivity (*p* = 0.005), overall survival (*p* = 0.021), and longer recurrence-free survival (*p* = 0.038). In recurrent disease, high expression of CXCL12 was also correlated with better overall survival (*p* = 0.040). Univariate and multivariate analysis revealed that high CXCL12 + tumor-infiltrating immune cells (TICs) (HR 0.99, *p* = 0.042, HR 0.99, *p* = 0.023, respectively) and combined CXCL12 + /CD66b + infiltration (HR 0.15, *p* = 0.001, HR 0.13, *p* = 0.001, respectively) are independent favorable predictive markers for recurrence-free survival.

**Conclusion:**

A high density of CXCL12 + TICs predicts a good response to chemotherapy, leading to a better overall survival and a longer recurrence-free interval. Moreover, with concomitant high CXCL12/CD66b TIC density, it is an independent favorable predictor of recurrence-free survival in patients with ovarian carcinoma.

**Supplementary Information:**

The online version contains supplementary material available at 10.1007/s00432-023-05466-8.

## Introduction

Ovarian carcinoma (OC) is the eighth leading cause of cancer-related death in adult women worldwide (Dinkelspiel et al. 2015; Webb and Jordan 2017) and has the highest mortality rate of all gynecological cancers (Bray et al. 2018; Sung et al. 2021). Nonspecific symptoms and stealth growth of the tumor often lead to diagnosis at a locally advanced stage of disease with metastasis to secondary sites (Badgwell and Bast 2007).

OC consists of a number of different histologic subtypes, of which high-grade serous OC (HGSC) is the most common (Matulonis et al. 2016). Complete cytoreductive surgery is the first-line treatment, usually followed by adjuvant platinum- and taxane-based chemotherapy (du Bois et al. 2005; Querleu et al. 2017). FIGO staging is the most important tool for determining further therapy and predicting prognosis (Javadi et al. 2016). Even after an initial good response to treatment, OC often relapses, develops chemotherapy resistance, and recurs with chemoresistant disease, leading to further lines of therapy with less benefit (Gupta et al. 2019). OC response to cytotoxic drugs is variable even among patients with the exact same FIGO stage and treatment approach. Biomarkers that can decipher this heterogeneity and predict chemosensitivity could aid in the adaptation of current therapeutic techniques and the identification of novel therapeutic targets with a higher odds of success. To date, only a few markers have made it into clinical practice (Guo et al. 2022; Ruscito et al. 2017; Steg et al. 2012).

Tumorigenesis is characterized by interactions between tumor cells and resident cells in the tumor microenvironment (TME) (Anderson and Simon 2020; Wang et al. 2017). The intricate interplay between cancer cells and non-neoplastic cells, the number and activation status of various immune cell types, and the expression of various immunomodulating substances determine the direction. A significant part of the cross-talk between these different cells is mediated by chemokines (Balkwill 2004; Nagarsheth et al. 2017). Similar to other chemokines, stromal cell-derived factor 1 (CXCL12), also known as CXCL12 (C–X–C Motif Chemokine Ligand 12), is defined through its substantial chemoattractant function to initiate cell migration through binding to specific GPCRs (G-protein-coupled receptors) (Legler and Thelen 2016; Parkin and Cohen 2001), particularly CXCR4 (C–X–C chemokine receptor type 4) and CXCR7 (C–X–C chemokine receptor type 7) (Teicher and Fricker 2010). Various signal transduction pathways promote cellular functions such as survival, proliferation, and gene expression when the receptors are activated (Kucia et al. 2004). These signals enable cells to regulate cytoskeletal dynamics, adhesion, and migration and ultimately to migrate along the chemogradient (Chen et al. 2018). Hematopoietic cell lines, such as lymphocytes, monocytes, and neutrophils, express CXCR4 most frequently, although it is also present in endothelial cells and malignant cells. All cells expressing functional CXCR4 on their surface can follow a CXCL12 gradient, including tumor cells, especially in organs known to be the most common sites of metastasis, such as the liver, bone marrow, and lung (Cojoc et al. 2013; Müller et al. 2001). Upon CXCL12 binding, CXCR4 is rapidly phosphorylated, internalized, and largely sorted into the degradation pathway, leading to downregulation of CXCR4 density, while CXCR7 enters the regeneration pathway and returns to the cell surface (Busillo and Benovic 2007; Uto-Konomi et al. 2013).

CXCL12, classified as either a homeostatic or inflammatory chemokine, is constitutively expressed in various organs or upregulated in tissues responding to physical or chemical agents (Jin et al. 2008; Teicher and Fricker 2010). In the TME, CXCL12 is mainly expressed by cancer-associated fibroblasts (CAFs), but endothelial cells and cancer cells can also produce CXCL12 (Orimo et al. 2005). It is thought to control and regulate various unique aspects of cancer in an autocrine and paracrine manner (Balkwill and Mantovani 2012; Grivennikov et al. 2010; Lin and Karin 2007). The hyperactivation of CXCL12/CXCR4 in cancer cells compared to their normal counterparts makes this axis a promising target for targeted therapy of cancer cells (Chatterjee et al. 2014; Scotton et al. 2001, 2002).

The endocrine CXCL12/CXCR4 axis is involved in the early phase of malignant transformation. Guo et al. showed that the CXCL12/CXCR4 signaling drives proliferation, migration, and invasion of ovarian cancer cells and may ultimately lead to ovarian cancer cell metastasis (Guo et al. 2014). Other studies showed that CXCL12 stimulates angiogenesis and reduces tumor cell apoptosis and tumor necrosis in OC (Kryczek et al. 2005; Righi et al. 2011). Research addressing the relationship between CXCL12 and tumor suppression shows that CXCL12 plays a role as a tumor-suppressive cellular brake. An experimental study in pancreatic cancer showed that CXCL12 interrupts the growth and metastasis of primary tumor through cell-cycle arrest, ultimately leading to an increase in overall survival (Roy et al. 2014). Similar beneficial effects of CXCL12 on clinical outcomes have been observed in patients with osteosarcoma and breast cancers (Baumhoer et al. 2012; Mirisola et al. 2009). In addition, a previous study showed that cisplatin resistance in OC is induced by CXCL12/-CXCR4 signaling by triggering epithelial–mesenchymal transition (EMT) processes (Zhang et al. 2020).

This study aimed to evaluate the expression of CXCL12 in primary and recurrent epithelial ovarian cancer tissue and explore whether its assignability to tumor/immune cells affects chemosensitivity or prognostic survival. Moreover, in our previous study, we identified CD66b + neutrophils as an independent predictor of better survival in OC (Posabella et al. 2020). Mixed immune cell infiltration, such as NLR (neutrophil-to-lymphocyte ratio) and NMR (neutrophil-monocyte ratio), has been repeatedly studied as an indicator of prognosis. We intended to examine an intratumoral collective of mixed neutrophilic and CXCL12 + TICs cell populations for synergistic effects.

## Materials and methods

### Patients

Formalin-fixed tissue specimens of primary ovarian carcinomas and associated recurrences were collected in collaboration between the Institutes of Pathology of the University Hospital of Basel and the Cantonal Hospitals of Baden, Liestal, and St. Gallen. To obtain a homogeneous cohort, we included only high-grade ovarian carcinomas after subtyping (G.S.) the carcinomas according to previous publications (Singer et al. 2002, 2003). Patients with neoadjuvant chemotherapy were not included. All patients underwent standard of care treatment, including initial debulking surgery and at least three cycles of platinum-based adjuvant chemotherapy. Subsequently, all patients suffered from recurrences. If the recurrence occurred within 6 months after cessation of chemotherapy, the disease was defined as chemoresistant. In contrast, the chemosensitive subgroup was characterized by progression-free intervals longer than 6 months. Cancer tissues from the recurrences were obtained by biopsies. Second-line therapy for initial recurrences consisted of multiple cycles of platinum-based chemotherapy depending on the response to chemotherapy. No patient received concomitant PARP inhibitors, ICI therapy or secondary debulking surgery (SDS). Individual clinical data were obtained from the medical records and gynecologic tumor registries of the participating institutions (U.G). The statement concerning the clinical data collection and ethical considerations can be found in previous publications (Stadlmann et al. 2008, 2006; Stadlmann et al. 2007a, b; Stadlmann et al. 2007a, b).

### Tissue microarray construction

The immunohistological analysis was performed using a tissue microarray (TMA) containing primary tumor samples and their matched recurrences from the same patients. The tissue microarray (TMA) for this study was available from our previous studies (Stadlmann et al. 2008, 2006; Stadlmann et al. 2007a, b; Stadlmann et al. 2007a, b). The construction of the TMA has been previously described (Sauter et al. 2003). Briefly, a total of 350 tissue cores, fixed in 4% buffered formalin and embedded in paraffin, are represented on the array. H&E-stained slides section was used on the donor block to define representative tissue regions and guide tissue cylinder punches (0.6 mm in diameter) and transferred in paraffin-wax blocks. The resulting TMA block was cut into 3-µm sections and arrayed on glass slides for further immunohistochemical staining (Simon et al. 2004; Stadlmann et al. 2007a, b).

### Study design

A total of 47 patients with primary high-grade serous carcinomas (HGSC) and their matched recurrence were entered into this study. Overall survival (OS), with a 3-year follow-up and recurrence-free survival (RFS), defined as 6 months progression-free-interval, were examined as clinical endpoints. The clinicopathological variables of patients of this cohort considered are FIGO stage, residual disease, number of chemotherapy cycles, and chemoresistance as presented in Table 1. This manuscript is written according to the REMARK guidelines (McShane et al. 2006).

**Table 1:** Patient characteristics (*n* = 47)

Characteristics	*n* = 47
Age (median, range)	58 (34–77)
FIGO stage (*n*, %)	
II	1 (2.1)
IIIA	1 (2.1)
IIIB	5 (10.6)
IIIC	32 (68.2)
IV	8 (17.0)
Residual disease (*n*, %)	
None	16 (34.0)
< 2 cm	17 (36.2)
> 2 cm	13 (27.7)
Numbers of chemotherapy cycles (*n*, %)	
< 6	7 (14.9)
6 or more	39 (83.0)
CS** (*n*, %)	33 (70.2)
CR** (*n*, %)	14 (29.8)
RFS*** (mean/SE)	10.1 (1.4)
OS*** (mean/SE)	41.4 (4.3)
CXCL12 TIC P (median/IQR)	35.4 (15.5*–*60)
CXCL12 TIC R (median/IQR)	41.2 (10*–*47)
CXCL12 Score P (median/IQR)	101.9 (50*–*200)
CXCL12 Score R (median/IQR)	120 (20*–*180)

Missing clinicopathological information was assumed to be missing at random

**CS chemosensitive, *CR* chemoresistant

***RFS recurrence-free survival; *OS* overall survival

### Immunohistochemistry (IHC) and visual analysis

Immunohistochemical analysis was performed using a standard indirect immunoperoxidase procedure (ABC Elite, Vectra Laboratories). Tissue-array sections were first dewaxed and rehydrated in distilled water and immersed in methanol using 0.5% hydrogen peroxide to limit non-specific background staining. To retrieve antigenicity, sections were immersed in 10% normal goat serum (DakoCytomation, Carpinteria, CA) and incubated with a primary antibody that was specific for CXCL12 (Abcam ab9797). Subsequently, the sections were incubated with secondary antibodies (DakoCytomation) and conjugated to a peroxidase-labeled enzyme. Visualization of the antigen was achieved by following the addition of 3-amino-9-ethylcarbazole plus substrate-chromogen (DakoCytomation) and counterstaining with Gill’s hematoxylin.

The evaluation was independently scored at 20 × magnification by two trained medical research fellows (P.K. and A.L.) and validated by our experienced pathologists (E.C. and G.S.) who were blinded to clinical and histopathological parameters. Our histoscore is based on the percentage of CXCL12 + tumor cells with clearly visible membranous/cytoplasmic staining x relative staining intensity. Staining intensity was scored as follows: 0 = negative, 1 = weak, 2 = moderate and 3 = strong. Tissue areas showing necrosis, staining artifacts or presenting < 50% of preserved tumor tissue were excluded from the analysis. In addition, an absolute quantity of CXCL12 + tumor-infiltrating immune cells (TICs) cells was assessed by counting positive (stained) cells in each region of interest (ROI). The absolute count assessment included tumor stroma and excluded intravascular cells from analysis.

### Statistical analysis section

The statistical analyses were made using STATA software version 13 (StataCorp, College Station, TX, USA). CD66b data were available from a previous publication [44]. We used cutoff scores to build two subgroups of CXCL12 + TICs with either low or high CXCL12 density. Cutoff scores for low or high density were defined using the 25th percentile, based on regression tree analysis. In primary carcinomas, the specific cutoff score was set at 10.5 positive cells/punch and 10 cell/punch in recurrences. Conclusive data for CXCL12 were available in 43 biopsies of primary and 41 biopsies of matched recurrent carcinomas, respectively. Cutoff scores used to classify ovarian carcinomas with low or high CXCL12 expression were defined according to the histoscore (0–300). Correlations among clinicopathologic features and CXCL12 tumor positivity and CXCL12 + TICs infiltration were calculated using Chi-Square, Fischer’s exact, and Kruskal–Wallis tests.

The univariate/multivariate Cox regression model was used to compute hazard ratios (HR) and 95% confidence intervals (CI) for known prognostic variables (age, residual disease, stage, and a number of chemotherapy cycles) and CXCL12 and separately with CXCL12/CD66b to determine effects on survival times. *p* values < 0.05 were considered statistically significant.

For survival analysis, the Kaplan–Meier curve was applied to estimate probabilities of RFS and OS and log-rank was tested for significance. The Cox proportional hazard assumption was checked for all markers by analyzing the correlation of Schoenfeld residuals and ranks of individual failure times. Any missing clinicopathological information was assumed to be missing at random.

Correlation analysis of CXCL12, CD66b, OX40, IL-17, CXCR4, pCXCR4, FoxP3, MPO, and IL-22 protein expression from our previous studies was calculated using the Spearman’s correlation test (Posabella et al. 2020; Ramser et al. 2018; Walther et al. 2022).

## Results

### Patients’ characteristics

Patients’ mean age at diagnosis was 58 (range 34–77). The recorded FIGO classification was the following; 1 patient FIGO stage II (2.1%), 38 patients FIGO stage III (80.9%), and 8 patients FIGO stage IV (17%). After initial surgery, 16 patients (34%) had no residual disease, 17 patients (36.2%) had optimal debulking (residual tumor < 2 cm), and 13 patients (27.7%) still had macroscopic disease with residual tumor > 2 cm. All patients received chemotherapy. However, while 33 tumors (70.2%) were chemosensitive, 14 were chemoresistant (29.8%). Of all patients, 39 received 6 or more chemotherapy cycles (83%). Median recurrence-free survival (RFS) was 10.1 months (SE 1.4) and overall survival (OS) was 41.4 months (SE 4.3). Clinicopathological characteristics are summarized in Table 1.

### CXCL12 expression in paired primary and recurrent ovarian carcinoma

**Fig. 1:**
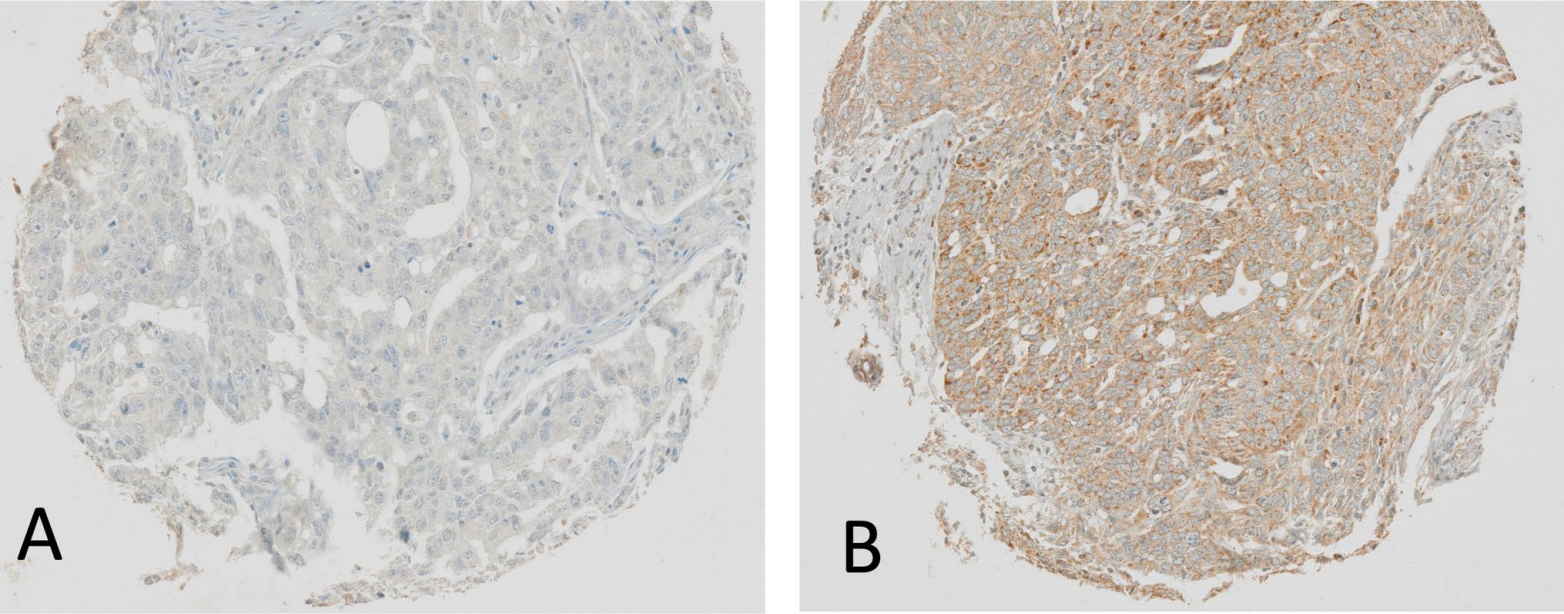
Example of low (**A**) and high (**B**) CXCL12 TIC expression; magnification 10x

CXCL12 immunohistochemistry demonstrated both cytoplasmic and membranous staining in positive cells. Representative pictures of tumors with low and high expression of CXCL12 are shown in Fig. 1.

Average count of CXCL12 + tumor cells in primary and recurrent cancer biopsies was 35.4 (per tissue punch) (± 28.59 SE) and 41.2 (± 47.14 SE), respectively (Table 1). In particular, CXCL12 was expressed to high extents (cutoff = 10.5 cells/punch in primary, cutoff = 10 cells/punch in recurrent cancer, respectively) in 32 out of 43 primary and in 29 out of 41 recurrent cancer biopsies (Table 2A, B). Mean histoscores for CXCL12 + expression in primary and recurrent OC biopsies were 101.9 (± 91.78 SE) and 120 (± 81.26 SE), respectively (Table 1).

**Table 2:** Patients’ characteristics according to dichotomized distribution of CXCL12-positive TIC in (A) primary cancer biopsies in the overall cohort (cutoff = 10.5 cells/punch, 25th percentile, *n* = 43) (B) recurrent cancer biopsies in the overall cohort (cutoff = 10 cells/punch, 25th percentile, *n* = 41)

(A)	CXCL12^high^, *n* = 32 (%)	CXCL12^low^, *n* = 11 (%)	*p*-value
Age (mean, range)	57 (34–77)	58.8 (39–69)	0.486
FIGO stage (*n*, %)			0.519
II	1 (3.1)	0 (0.0)	
IIIA	0 (0.0)	0 (0.0)	
IIIB	5 (15.6)	0 (0.0)	
IIIC	21 (65.6)	8 (72.7)	
IV	5 (15.6)	3 (27.3)	
Residual disease (*n*, %)			0.411
None	11 (34.4)	3 (27.3)	
< 2 cm	13 (40.6)	3 (27.3)	
> 2 cm	7 (21.9)	5 (45.4)	
Numbers of chemotherapy cycles (*n*, %)			
< 6	4 (12.5)	2 (18.2)	0.667
6 or more	27 (84.4)	9 (81.8)	
CS** (*n*, %)	26 (81.3)	4 (36.4)	0.005
CR** (*n*, %)	6 (18.7)	7 (63.6)	
RFS*** (mean/SE)	11.2 (3.60)	5.3 (2.26)	0.038
OS*** (mean/SE)	48.1 (9.35)	29 (7.14)	0.021

(B)	CXCL12^high^, *n* = 29 (%)	CXCL12^low^, *n* = 12 (%)	*p*-value
Age (median, range)	56.2 (34–76)	61.4 (49–69)	0.08
FIGO stage (*n*, %)			0.528
II	1 (3.4)	0 (0.0)	
IIIA	1 (3.4)	0 (0.0)	
IIIB	5 (17.2)	0 (0.0)	
IIIC	17 (58.6)	9 (75.0)	
IV	5 (17.2)	3 (25.0)	
Residual disease (*n*, %)			0.75
None	10 (34.5)	5 (41.7)	
< 2 cm	11 (37.9)	3 (25.0)	
> 2 cm	7 (24.1)	4 (33.3)	
Numbers of chemotherapy cycles (*n*, %)			
< 6	5 (17.2)	1 (8.3)	0.44
6 or more	23 (79.3)	11 (91.7))	
CS** (*n*, %)	23 (79.3)	6 (50.0)	0.061
CR** (*n*, %)	6 (20.7)	6 (50.0)	
RFS*** (mean/SE)	10.9 (3.43)	6.5 (2.76)	0.134
OS*** (mean/SE)	49.1 (9.66)	29.5 (7.93)	0.004

Percentages may not add to 100% due to missing values of defined variables, missing clinicopathological information was assumed to be missing at random. Variables are indicated as absolute numbers, %, median or range. Age, RFS, and OS were evaluated using the Kruskal–Wallis test. FIGO stage, residual disease, numbers of chemotherapy cycles, and chemoresistance were analyzed using the Chi-Square or the Fisher’s exact test

**CS chemosensitive, *CR* chemoresistant

***RFS recurrence-free survival, *OS* overall survival

In primary cancer biopsies, we found a significant association between high density of CXCL12 + tumor-infiltrating immune cells and improved response to chemotherapy (*p* = 0.005). In addition, high expression of CXCL12 correlated significantly with better OS and RFS in primary group, and only with OS in the group of the recurrent cases (Table 2A, B).

Interestingly, no other parameter—including age, clinical stage, residual disease, or the number of chemotherapy cycles—was significantly correlated with CXCL12 expression in primary or recurrent tumors. Details on the distribution of dichotomized CXCL12 expression in association with clinicopathological data are reported in Table 2A, B.

### Kaplan–Meier curves

**Fig. 2:**
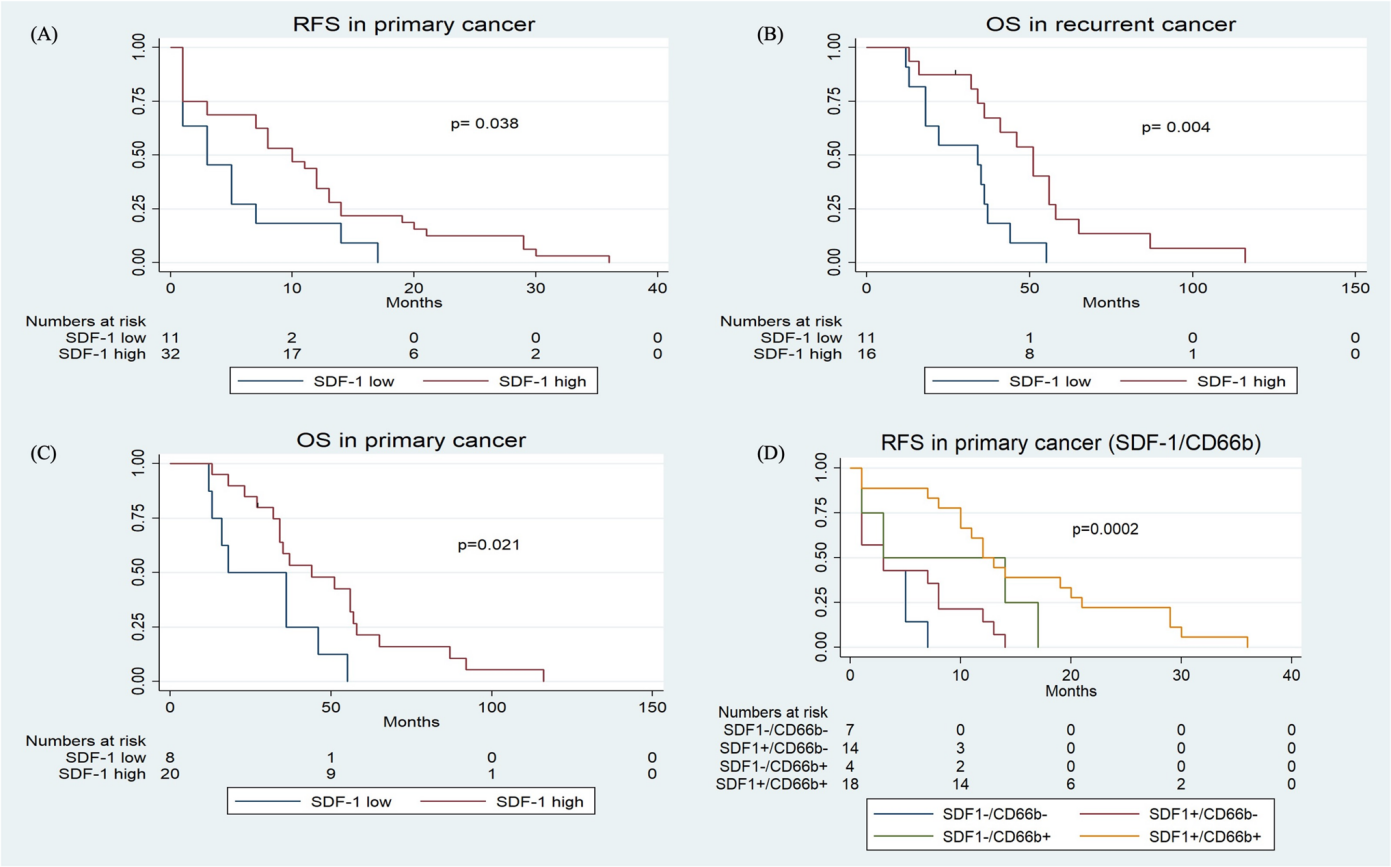
Kaplan–Meier survival curve of **A** recurrence-free survival according to CXCL12 TICs expression in primary cancer biopsies, **B** overall survival according to CXCL12 TICs expression in primary cancer biopsies, **C** overall survival according to CXCL12 TICs expression in recurrent cancer biopsies, **D** of recurrence-free survival according to CXCL12 TICs and CD66b expression in primary cancer biopsies. Kaplan–Meier survival curves were split according to CXCL12 + expression in patients bearing high-grade ovarian carcinoma as indicated. Blue line indicates tumors with low CXCL12 + expression. Red line refers to tumors with high CXCL12 + expression. **D** Cumulative effects of tumor infiltration by CXCL12 + and CD66b + cells were explored on recurrence-free survival. Blue line indicates tumors with low CXCL12 + and low CD66b + expression. Green line refers to tumors with high CD66b + expression. Brown line refers to tumors with high CXCL12 + expression and orange line refers to tumors with high CXCL12 + and high CD66b + expression

To assess the prognostic significance of CXCL12 expression, Kaplan–Meier survival curves were constructed. RFS and OS of patients with primary OC with strong CXCL12 expression were significantly improved, as compared with OC patients with weak CXCL12 expression (*p* = 0.038 and *p* = 0.021, respectively) (Fig. 2A, C).

Log-rank statistical analysis corroborated that strong CXCL12 expression, (of both tumor and TICs), was significant prognostic indicators for overall patients’ survival in recurrent cancers (*p* = 0.004) (Fig. 2B).

### Univariate and multivariate analysis of CXCL12 expression by tumor cells and tumor-infiltrating immune cells

When all clinicopathological parameters and CXCL12 + expression were tested by univariate analysis, a high expression of CXCL12 was found to be a prognostic marker for RFS for the entire cohort (HR 0.99; 95% CI 0.98*–*1.00; *p* = 0.042). Moreover, as expected, residual disease and number of chemotherapy cycles were both significantly associated with a poor prognosis in univariate analysis (HR 3.67; 95% CI 1.62*–*8.31; *p* = 0.002, HR 1.28; 95% CI 1.05*–*1.55; *p* = 0.013) (Table 3A).

**Table 3:** Univariate and multivariate hazard Cox regression analysis of recurrence-free survival (A) CXCL12 + TIC infiltration and (B) CXCL12 + /CD66b expression

(A)	Univariate			Multivariate		
	HR	95% CI	*p* values	HR	95% CI	*p* values
CXCL12 + TIC infiltration	0.99	0.98–1.00	0.042	0.99	0.97–1.00	0.023
Age	1	0.97–1.03	0.888	1	0.98–1.04	0.787
Residual disease < 2 cm	1.13	0.56–2.28	0.724	1	0.44–2.24	0.996
Residual disease > 2 cm	3.67	1.62–8.31	0.002	3.6	1.32–9.75	0.012
N of chemotherapy cycles	1.28	1.05–1.55	0.013	1.43	0.54–3.83	0.473
FIGO IIIA	0.34	0.02–5.72	0.455			
FIGO IIIB	0.93	0.11–8.04	0.944	2.3	0.22–24.27	0.488
FIGO IIIC	1.21	0.16–9.03	0.851	1.48	0.18–12.49	0.715
FIGO IV	1.48	0.18–11.94	0.712	1.46	0.17–12.83	0.734

(B)	Univariate			Multivariate		
	HR	95% CI	*p* values	HR	95% CI	*p* values
CXCL12 + /CD66b-	0.55	0.21–1.43	0.222	0..58	0.20–1.64	0.302
CXCL12-/CD66b +	0.29	0.07–1.10	0.071	0.27	0.07–1.08	0.064
CXCL12 + /CD66b +	0.15	0.05–0.43	< 0.001	0.13	0.04–0.42	0.001
Age	1	0.97–1.03	0.888	1	0.96–1.03	0.799
Residual disease < 2 cm	1.13	0.56–2.28	0.724	1.07	0.48–2.37	0.868
Residual disease > 2 cm	3.67	1.62–8.31	0.002	3.44	1.25–9.44	0.016
N of chemotherapy cycles	1.28	1.05–1.55	0.013	1.18	0.43–3.25	0.746
FIGO IIIA	0.34	0.02–5.72	0.455			
FIGO IIIB	0.93	0.11–8.04	0.944	2.35	0.25–22.19	0.456
FIGO IIIC	1.21	0.16–9.03	0.851	2.54	0.28–22.94	0.405
FIGO IV	1.48	0.18–11.94	0.712	1.66	0.18–15.51	0.657

Univariate and multivariate analyses showing hazard ratios and p value for all primary cancer biopsies (*n* less than 43 due to missing values) (A) conferred by categorized CXCL12 TICs expression, age, residual disease after cytoreductive surgery, number of chemotherapy cycles and FIGO classification, (B) conferred by categorized CXCL12/CD66b expression, age, residual disease after cytoreductive surgery, number of chemotherapy cycles and FIGO classification

Multivariate hazard Cox regression confirmed the potential of CXCL12 + expression as prognostic indicators of favorable RFS (HR 0.98; 95% CI 0.97*–*1.00; *p* = 0.023). Besides, solely macroscopic residual disease maintained its role as an independent prognostic factor of poor prognosis (HR 3.59; 95% CI 1.32*–*9.75; *p* = 0.012) (Table 3A).

### Combined analysis of CXCL12 and CD66b expression

In a previous study, we had reported an association between neutrophil infiltration and chemosensitivity in OC (Posabella et al. 2020). Thus, we grew curious to verify whether CXCL12 expression and CD66b + neutrophil infiltration were of synergic prognostic significance.

Again, Kaplan–Meier plots were used to illustrate survival rates in primary OC. The RFS survival rates were significantly different depending on the pattern of immune infiltration (Fig. 2D). Simultaneous strong CXCL12 + expression and CD66b + infiltration correlated with better RFS whereas absence of CXCL12 + expression and CD66b + infiltration indicated inferior RFS (*p* < 0.001).

Univariate and multivariate Cox regression analysis was repeated with combined categorized CXCL12/CD66b expression/infiltration. Again, CXCL12/CD66b positivity was confirmed to represent an independent favorable RFS predictor in primary cancer biopsies (HR 0.15; 95% CI 0.05*–*0.43; *p* =  < 0.001, 95% HR 0.13; CI 0.04*–*0.42; *p* = 0.001, respectively) (Table 3B).

### Spearman’s correlation analysis of CXCL12 and markers of the microenvironment

We used data from prior research to perform Spearman's correlation analysis in primary and recurrent OC to better understand tumor microenvironment (TME) as related to potential co-regulation of particular biomarkers. We included a panel of different immune markers and considered correlations above 0.35 to be relatively strong, correlations between 0.15 and 0.35 to be moderate, and those below 0.15 to be weak. The analysis showed the following significant correlations: in primary OC, CXCL12 + TICs expression showed a strong correlation with OX40 (rho = 0.512; *p* = 0.006) but not with CXCR4 and pCXCR4. In recurrent OC, CXCL12 + expression strongly correlated with CXCR4 and pCXCR4 (rho = 0.438; *p* = 0.036, rho = 0.515; *p* = 0.012) (Table S).

## Discussion

The poor clinical outcome of OC despite debulking surgery and adjuvant chemotherapy prompted a thorough investigation of the factors that might influence disease progression. The dismal prognosis is mainly based on the fact that about 70–80% of patients will relapse very soon after therapy (Pignata et al. 2017). Treatment of recurrence is an important issue, and long-term chemotherapy vs. re-operation is still controversial (Griffiths et al. 2011). The prognosis in recurrence is mainly determined by the chemosensitivity of the tumor. Patients who relapse during first-line treatment (refractory) or in the following months after (resistant) represent a very heterogeneous group with different biological tumor behaviors (Poveda et al. 2011). In this context, it is important to highlight the high toxicity of these therapies, which severely affects patients` quality of life, especially if the treatment is ultimately ineffective. Therefore, it is crucial to prolong relapse-free survival or predict chemosensitivity based on certain characteristics of the tumor microenvironment. In this study, we explored whether CXCL12 tumor expression or the CXCL12 + TICs infiltration predicts chemosensitivity and impacts survival.

The impact of CXCL12 signaling on ovarian cancer progression depends on the delicate balance between its ability to summon and activate immune cells vs. its ability to cause pathological conditions in a tumor (Chen et al. 2018; Fucikova et al. 2021). In a meta-analysis on cancer prognosis, high CXCL12 expression was associated with reduced absolute survival in patients with esophagogastric, pancreatic or lung cancer whereas the opposite was true in breast cancer patients. In colorectal and ovarian cancers, no statistical association with overall survival was found. However, in OC, particularly in the study with the longest follow-up and the largest cohort, the chorus persisted that moderate or high CXCL12 expression correlated with reduced disease-specific survival (Popple et al. 2012; Samarendra et al. 2017). In comparison to the retrospective meta-analysis, a prospective study by D’Alterio et al. found that high epithelial CXCL12 in OC was inversely related to PFS and OS, but significance was lost due to adjustment by overfitting. Interestingly, alternative staining pattern for epithelial and stromal CXCL12 expression showed different effects on survival. Although not significant, stromal expression showed a meaningful trend toward a survival advantage (D'Alterio et al. 2022). As a result of this study, high CXCL12 tumor expression (CXCL12 histoscore) was shown to correlate with better patient survival (*p* = 0.040). This finding, obtained only in the recurrent cancer group, failed to provide convincing data to either refute or confirm the literature, adding that the results obtained in the aforementioned studies were based on primary OC. The survival advantage in our cohort due to the high endogenous expression of CXCL12 in the recurrent group may be due to the inability of ovarian cancer cells to migrate and metastasize. Several theories for these differences in data obtained in different cancer types are found in the literature.

The results may reflect clinical biology or methodological differences, as CXCL12 expression varies in pattern among individual cancer types (Poveda et al. 2011). CXCL12 is thought to have a concentration-dependent bifunctional effect on numerous cell types. It has been described that cancer associated with metastasis disease like breast cancer may rely on downregulation of CXCL12 to advance to ectopic sources, as they may be more susceptible to chemoattraction when turned off (Wendt et al. 2008; Yu et al. 2017). In OC, significantly lower epithelial CXCL12 expression was found in stage IV as compared to stage III patients (Samarendra et al. 2017). Similarly, to breast cancer, high chemokine expression and positive survival outcome in OC might be closely related to local promotion and direct metastatic seeding of tumor cells into the peritoneal cavity (Yu et al. 2017). Another possible explanation is that CXCL12 has an impact on the biological behavior of cells in the TME, which could explain the diverging results (Guo et al. 2016). While cytoplasmic staining of the tumor is simultaneously indicative of its primary source, stromal production may influence cancer progression in different ways (Simon and Salhia 2022). Attracting cells at low CXCL12 concentrations and repelling cells at higher CXCL12 concentrations may explain the lack of extensive infiltration by T cells and immune surveillance in some organs (Poznansky et al. 2000). The microscopic CXCL12-rich cancer stroma may create immune-privileged sites when expressed intratumorally. By keeping CXCR4-expressing T cells away from the juxtatumoral compartment, the tumor escapes recognition and destruction by tumor-specific lymphocytes (Kohli et al. 2022).

In this study, we demonstrated that high CXCL12 + TICs correlated with better RFS in primary OC, independent of known risk factors. Also, high CXCL12 + TICs showed prognostic significance and were associated with better OS in the primary and recurrent groups. There is limited information in the cancer literature comparing CXCL12 TICs as biomarker with our results. For example, in colorectal cancer, where the role of CXCL12 expression and its association with the overall survival is controversial, our team showed that high expression of CXCL12 can increase the prognostic value of CD8 + T cell density in stage III disease (Lalos et al. 2021). In contrast, the results of Seo et al. suggested that CXCR4/CXCL12 blockade leads to CD8 + T-cell-mediated antitumor activity in pancreatic cancer (Seo et al. 2019). Another study independently confirmed that CXCR4 antagonism with plerixafor (AMD3100) enhances T cell infiltration by releasing trapped CXCR4-expressing T cells in CXCL12-rich stroma before reaching carcinoma cells (Feig et al. 2013). To better interpret the pattern of immune infiltrates besides their total number, the concept of the tumor immune phenotype (TIP) was introduced (Hornburg et al. 2021). Its strengths lie in considering the spatial distribution of T cells in the TME, and three TIPs have been proposed. In the immune-excluded phenotype, T cells accumulate in the tumor stroma, in the inflammatory-infiltrated phenotype, T cells accumulate in the epithelium and in the immune-desert phenotype; T cells are either absent or present in low numbers (Hegde et al. 2016). The spatial distribution, namely immune exclusion, in which T cells accumulate in the stroma, was associated with the worst clinical outcome in ovarian carcinoma (Desbois et al. 2020). Moreover, the study of Hornburg et al. found that spatial distribution facilitated further recruitment of T cells (Hornburg et al. 2021). The OC of the infiltrated phenotype is enriched with a specific subtype of cancer-associated fibroblasts (IL1 CAF). IL1 CAF-derived CXCL12 interacted almost exclusively with cognate CXCR4-expressing immune cells, particularly with CD8 + T cells, suggesting an important role of source dependent effects of CXCL12.

In our correlation analysis of immune markers, CXCL12/OX40 correlated strongly with FOXP3. In the recurrence group, the expression of CXCL12 + TICs correlated strongly with CXCR4 and pCXCR4. Based on the immune signature, we hypothesized that the immune configuration in this cohort's TME is slightly shifted toward CD4 + /CD8 + T cells. In addition to TIL density and spatial distribution, the TME polarization is associated with better OS (Zhang et al. 2003).

It has been repeatedly described that Treg cells characterized by the expression of FOXP3 inversely correlate with patient survival (Worzfeld et al. 2017). CD3 + /CD8 + lymphocytes and T-helper cells contribute to the antitumor response in OC (Goode et al. 2017). We hypothesize that high numbers of CD8 + polarized T cells, which preferentially infiltrate tumor epithelium, may have contributed to the survival advantage in this cohort. Furthermore, the bidirectional decision of migrating cells depends on homogeneous receptor/ligand interaction. We hypothesize that a strong correlation of CXCL12/CXCR4 and CXCL12/pCXCR4 in recurrent carcinoma will arrest migratory immune cells. A discrepancy in primary carcinoma may indicate an older migrated immune population or an interaction with CXCR7.

CAF-mediated CXCL12 expression is critically involved in epithelial–mesenchymal transition (EMT), which enables cancer cells to acquire cancer stem cell (CSC) phenotype and tumorigenicity. Recent studies demonstrate that CSCs can develop resistance to chemotherapy, and thus may be responsible for disease residuals and recurrence (Zhang et al. 2020). Interestingly, in our cohort, high TICs expression of CXCL12 rather than cancer-related CXCL12 expression predicts a good response to chemotherapy (*p* = 0.005). In addition to the cytotoxic effects of conventional anticancer therapies, therapeutic efficacy also involves a substantial immunological component. These off-target effects can either boost immune effector cells or reset the TME immune configuration by inhibiting or depleting immunosuppressive cells (Galluzzi et al. 2020). Chemotherapy with off-target effects could help to alter the immune configuration in favor of more immune vigilant cells, as the recurrent OC group in this study had higher CXCL12 expression and stronger TIC’s infiltration. These results could indicate time-dependent effects of CXCL12 expression, with a scheduled higher CXCL12 expression after chemotherapy being a favorable condition for prolonged survival.

In a recent study, we demonstrated that CD66b + can be assigned to an activated, immunovigilant subtype of neutrophils associated with longer RFS in OC (Posabella et al. 2020). Compared with other immune cells, the role of neutrophils in the TME is relatively poorly understood. The intratumoral neutrophil-to-lymphocyte ratio (iNRL) is less well documented than the peripheral NLR, an indicator of a poor prognosis in various cancers (Guthrie et al. 2013). Results in non-small cell lung carcinoma (nSCLC) showed that high iNRL correlated with poor OS (Ilie et al. 2012). Our observation is consistent with these findings, that a low neutrophil ratio has an impact on longer RFS. It is noteworthy that our crudely estimated neutrophil-to-lymphocyte ratio is imprecise and based on cutoff scores used for high/low biomarker expression, but it highlights the importance of the different ratio of immune cell subsets in the TME.

Despite focusing on the most common subtype of ovarian cancer (HGSOC), our study encountered limitations in terms of the availability of cancer tissues, constraints on consistent treatment options, and cases that were clinically suitable. As a result, our cohort size remained small, which constitutes the primary limitation of our research. These results should be confirmed in larger cohorts and prospective longitudinal studies additionally assessing the source of SDF-1. Furthermore, we were unable to clearly identify the cellular source of CXCL12. In the literature, CAF-derived CXCL12 has been described to coat cancer cells, whereas immunohistochemical approaches of most studies indicate tumor cells as the primary source of CXCL12 (Biasci et al. 2020). Differentially assessed expression patterns of CXCL12, inadequate analysis of CXCL12 in relation to its receptor, and the inaccurate source of CXCL12 within the tumor may explain the results obtained in our study which differ to existing literature. Therefore, a limitation is probably our immunohistochemical approach, with poor specificity of many antibodies and different rules for interpretation of expression, which reduces comparability to similar studies in other tumors. Our technical results remain to be further verified by other experimental methods to characterize regulation mechanisms like the silencing of CXCL12 in ovarian carcinoma. Expanded research with a larger cohort would contribute to a more unified insight on CXCL12 as a biomarker.

## Conclusion

In conclusion, the present exploratory study highlights that high CXCL12 TICs in OC are an independent positive predictive marker for chemosensitivity, OS, and RFS in primary tumors and prognostic for better overall survival in recurrent carcinoma. Our data suggest that the combined CXCL12/CD66b TICs plays a critical role in recurrence-free survival in OC, possibly indicating an effective synergistic immune response. Expression of CXCL12 + TICs may be a potential predictive marker for sensitivity to chemotherapy, whereas CXCL12 + tumor expression showed no association with prognosis.

## Supplementary Information

Below is the link to the electronic supplementary material.

Supplementary file1 (DOCX 367 KB)

## Data Availability

Raw data can be obtained by contacting the corresponding author.
