# Supplementary material for: High density of CXCL12-positive immune cell infiltration predicts chemosensitivity and recurrence-free survival in ovarian carcinoma
Source: J Cancer Res Clin Oncol. 2023 Nov 15;149(20):17943–55. doi: 10.1007/s00432-023-05466-8 (PMC10725329; doi:10.1007/s00432-023-05466-8)
Supplement: Supplementary file 1 — Supplementary file1 (DOCX 367 KB) [file 432_2023_5466_MOESM1_ESM.docx]

Supplementary tables


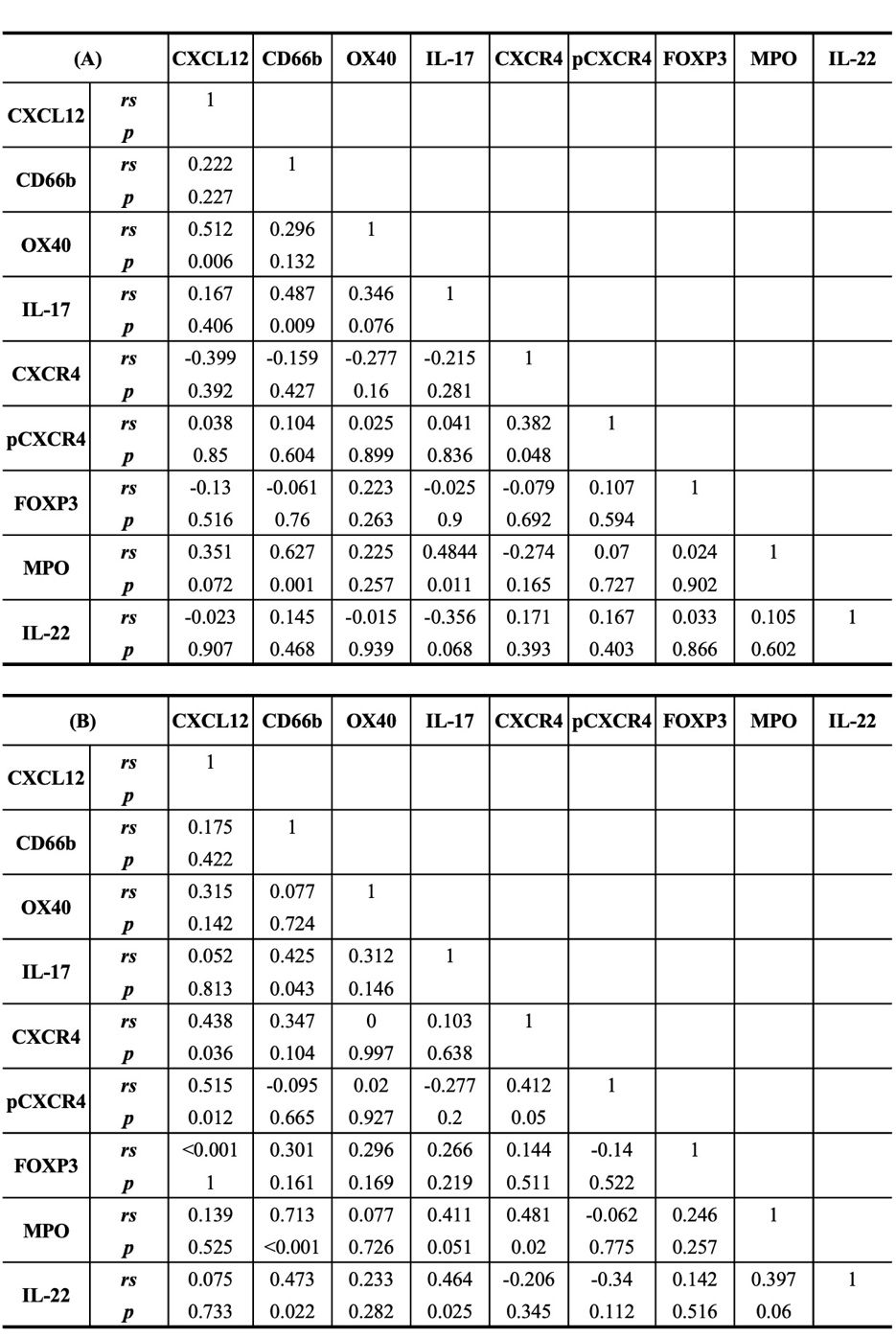
Table S: Correlation analysis of CXCL12, CD66b, OX40, IL-17, CXCR4, pCXCR4, FOXP3 MPO and IL-22 positive tumor immune cell infiltration in A) primary ovarian carcinoma B) recurrent ovarian carcinoma

Correlation analysis CXCL12, CD66b, OX40, IL-17, CXCR4, pCXCR4, FoxP3 MPO and IL-22 showing rho and p-value
